# Infection associated acute interstitial nephritis; a case report

**DOI:** 10.15171/jnp.2017.09

**Published:** 2016-10-25

**Authors:** Rupesh Raina, Shirisha Ale, Tushar Chaturvedi, Luke Fraley, Robert Novak, Natthavat Tanphaichitr

**Affiliations:** ^1^Division of Nephrology, Department of Internal Medicine and Research Cleveland Clinic Akron General, Akron, Ohio, USA; ^2^Northeast Ohio Medical University, Rootstown, Ohio, USA; ^3^Department of Pathology & Lab services, Akron Children’s Hospital, Akron, Ohio, USA

**Keywords:** Acute interstitial nephritis, Infection associated AIN, Drug induced AIN, Dental abscess

## Abstract

**Background:**

Acute interstitial nephritis (AIN) is a clinico-pathological syndrome associated with a variety of infections, drugs, and sometimes with unknown causes. It is a common cause of acute kidney injury (AKI) and subsequent renal impairment, which often times is under-diagnosed. Infection-associated AIN occurs as a consequence of many systemic bacterial, viral, and parasitic infec-tions; however, its incidence has decreased significantly after the advent of antimicrobials. Infection-associated AIN presents with both oliguric or non-oliguric renal insufficiency, without the classical clinical triad of AIN (fever, rash, and arthralgia). In this scenario the renal function is usually reversible after the infection is treated. In most cases, patients with acute renal failure present with extra-renal manifestations typically detected in underlying infections. Renal biopsy serves as the most definitive test for both the diagnosis and prognosis of AIN.

**Case Presentation:**

In this paper, we will address one such case of biopsy-proven AIN. In this case, the patient presented with severe AKI induced by anaerobic streptococcus, leading to a periodontal abscess, which was successfully treated with corticosteroids and requiring renal replacement therapy (RRT).

**Conclusions:**

AIN should be considered in the differential for unexplained AKI. Initial management should include conservative therapy by withdrawing any suspected causative agent. Renal biopsy is needed for confirmation in cases where kidney function fails to improve within 5–7 days on conservative therapy. Risk of immunosuppression is very important to consider when giving steroids in patients with infection induced AIN, and steroids may have to be delayed until the active infection is completely controlled.

**Implication for health policy/practice/research/medical education:**

Infection-associated AIN presents with both oliguric and non-oliguric renal insufficiency, without the classical clinical triad of AIN (fever, rash, and arthralgia).

## 1. Introduction

In 1898, Councilman first described infection-associated AIN in association with streptococcal and diphtheria infections (1). It was considered the first studied type of AIN. This case of AIN result from an immune reaction against renal interstitial endogenous antigens through cell-mediated immune mechanisms and also hapten-mediated mechanism in some infections (2,3).

Its incidence has decreased due to widespread use of antibiotics, and accounts for 5%-10% of all AIN cases, but with recent advancement of early diagnostic techniques it is becoming more common (1-3). Its true incidence is often underestimated as suspected patients with clinical evidence are not always subjected to renal biopsy for confirmation. In elderly patients and patients with severe acute kidney injury (AKI) and multiple comorbidities, empirical treatment is preferred, and milder cases are usually under detected due to absence of classic clinical symptoms (2).

Infection-associated AIN presents with acute renal failure without the classic triad of AIN (pyrexia, arthralgia, rash) along with specific clinical features of underlying infection (3). Eosinophiluria and proteinuria are not specific and their absence does not exclude its possibility (4). Many recent studies found that the classic clinical triad of fever, rash, and arthralgia is seen only in 10%-40% of all AIN cases (5) suggesting the importance of renal biopsy for definitive diagnosis. Prognosis and long term renal complications of AIN can be estimated based on severity and extent of inflammatory damage on renal biopsy findings. Factors associated with poor renal recovery include longer period of AKI, elderly age group, along with biopsy findings showing severe diffuse interstitial damage in the form of tubular atrophy with granulomatous interstitial lesions and interstitial fibrosis (2,6,7).

Management of infection-associated AIN often involves supportive therapy and treating the underlying infection, which usually results in complete recovery of renal function. The role of steroids in the treatment of AIN is reported to be beneficial by few retrospective studies and many anecdotal reports, however evidence from randomized studies is still lacking (3,8,9).

## 2. Case Presentation

A 56-year-old Asian woman was brought to the emergency department with a history of nausea, vomiting, poor oral intake, and decreased urination for 3-4 days. Past medical history was significant for type-2 diabetes mellitus and hypertension, well-controlled on metformin and lisinopril respectively. Patient was afebrile and hemodynamically stable and, denied any fever, rash, joint pains, cough, abdominal pain, difficulty urination, or discoloration of urine. Physical exam was normal with clear lungs, normal heart sounds, soft non-tender abdomen without organomegaly, clear skin, and normal joints. Initial work-up was significant for elevated white blood cell count (WBC) of 10.1/mm, blood urea nitrogen (BUN) of 56 mg/dL, and creatinine of 7.4 with electrolytes within normal range. Urinalysis showed 2+ proteinuria, sterile pyuria with 7-8 white blood cells and eosinophils positive for Hensel’s stain. The patient was admitted and supportive treatment for AKI was started. By second day, her condition deteriorated with further increase in serum creatinine along with severe drop in urine output. The patient developed severe respiratory distress from fluid overload and pulmonary edema leading to acute respiratory failure, requiring institution of mechanical ventilation and continuous renal replacement therapy (CRRT).

Lab tests performed to discover the etiology of the AKI showed low C3, normal C4, and positive ANA (1:80 titer). Other tests including C-ANCA, P-ANCA, liver function tests, hepatitis panel, anti streptolysin O (ASO) titers, peripheral smear, and blood cultures were normal. Renal ultrasound and transesophageal echocardiogram (TEE) showed normal findings. Meanwhile, renal function and fluid status began improving on RRT, and her respiratory distress resolved.

The patient was found to have a raised WBC to 16.7 with predominant neutrophils, and empiric treatment for infection with vancomycin and piperacillin+ tazobactam started.

The patient described a mild jaw pain that had been persistent for more than a week, upon examination she was found to have a tender dental abscess. She clearly denied use of any over-the-counter pain pills or antibiotics, and imaging revealed a periodontal abscess with infected tooth, which was drained along with extraction of the tooth. Cultures from the abscess grew anaerobic streptococci and the patient was started on clindamycin.

**Figure 1:**
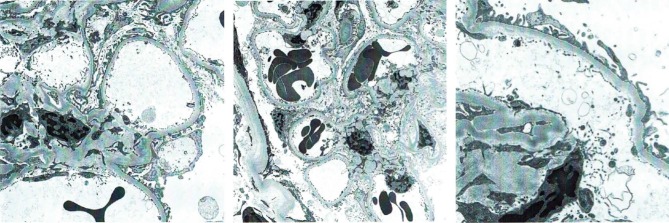
(A) Ten micron electron microscopic picture of kidney biopsy showing patchy inflammatory changes (B,C) 2 micron electron microscopic picture of kidney biopsy showing cellular infiltration.

A renal biopsy was performed, suspecting AIN secondary to streptococcal infection as the likely cause of AKI. Histopathology revealed patchy interstitial inflammatory infiltrates with prominent eosinophilic component most consistent with AIN (Figure 1).

**Figure 2:**
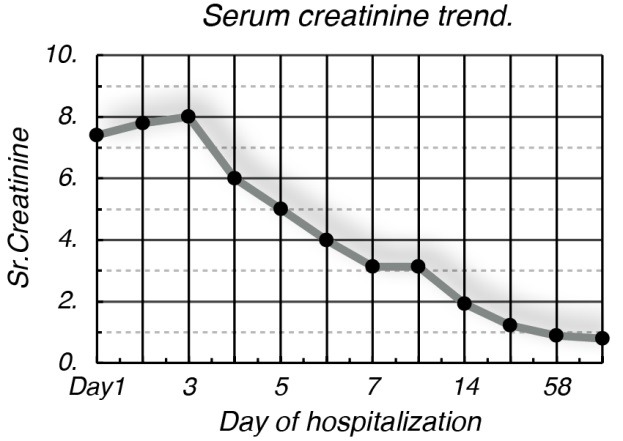
Serum creatinine during the days of hospitalization.

**Table 1:** Retrospective studies on use of steroids for AIN

**Study**	**Galpin et al**	**Buysen et al**	**Muriithi et al**	**Clarkson et al**	**González et al**
No. of patients	14	27	133	42	61
Steroid group	8	10	114	16	52
Dosage & duration of steroid	Prednisone oral 60 mg/day for 10 days	Methylprednisone IV 3 g pulse Tx 3 days	Initially IV followed by oral 60 mg for 7.5 weeks	Methylprednisone IV 500 mg pulse therapy 3 days	Dose and duration variable
Outcome in steroid group. - % of complete recovery - Duration of recovery - Final Serum Cr	- 75% - Mean 30 days - Mean final Serum Cr 1.43	- 60% - Faster recovery (NS)	- 47% - 12 weeks - Mean final Serum Cr 1.4	No significant difference	- 54% (96.4 % dialysis independent) - Mean 13 days - Mean final Serum Cr 2.1
Outcome in conservative treatment group - % of complete recovery - Duration of recovery - Final Serum Cr	- 33% - Mean 84 days - Mean final Serum Cr 1.9	NA	NA	No significant difference	33% (55.6% dialysis independent) - Mean 34 days - Mean final Serum Cr 3.4

Abbreviations: NA, not applicable; NS, not significant.

Oral prednisone with 60 mg per day was immediately initiated on the seventh day of hospitalization, and renal function steadily improved with eventual discontinuation of dialysis. Patient was discharged on 4 weeks of maintenance dose of prednisone and the dose was tapered thereafter. At her following clinic visits in 4 and 8 weeks, renal function had normalized with complete resolution of proteinuria. With no other obvious etiology for AIN, after excluding the possibility of drugs, we concluded that streptococcal dental infection was the culprit of AIN, and the treatment with steroids helped in complete recovery from renal failure.

## 3. Discussion

Infection-associated AIN is likely to develop during the course of many systemic infections from bacterial, viral, and parasitic organisms (3). It has a variable clinical presentation depending on the causative organism along with renal impairment ranging from mild self-limiting renal dysfunction to progressive renal impairment resulting in chronic kidney disease (CKD) (5). Laboratory results are usually nonspecific. Renal biopsy showing interstitial edema and predominant lymphocytic interstitial infiltrates remains the definitive test for diagnosis for any type of AIN (2,6). Infection-associated AIN show prominent neutrophilic infiltration and tends to be negative on immunofluorescence microscopy (3). Extensive interstitial damage can result in irreversible tubulointerstitial fibrosis leading to progression of AKI into CKD (3,10).

Basic principles of management in infection-associated AIN are similar to other cases of AKI which is mainly renal supportive therapy with a special focus on limiting the inflammatory damage by controlling the causative infection and by achieving immunosuppression (2,6). Steroid therapy started early in the course likely limits the inflammatory cellular infiltration and edema thereby preventing the fibrosis and scarring, and promoting faster recovery of renal function without long term complications (3-5,8,11).

Some retrospective studies and several anecdotal reports support the use of steroids for faster recovery with better prognosis in patients with AIN. Galpin et al (12) in 1978 studied the benefits of treating AIN with steroids in their non-randomized study. Around, 8 of 14 patients were treated for 10 days with steroids showing lower final serum creatinine(1.4 vs 1.9), shorter duration of renal dysfunction, and greater percentage of patients returning to baseline renal function (75% vs 33%) in the steroid group. A multi-center retrospective study on 61 AIN patients by González et al found that the use of steroids is beneficial in lowering serum creatinine significantly and achieving independence from dialysis sooner (8). In this study, 52 patients received steroids and 9 were conservatively treated. Final serum creatinine levels in the steroid treatment group were much lower (mean serum creatinine 2.1 vs 3.5) compared to the conservative treatment group. Even though they did not use a uniform dosage or duration of steroid therapy, they noticed better recovery of renal function with earlier initiation of steroids (13 vs 34 days) (8).

A study by Buysen et al (5) looked at 27 AIN patients among which 9 were secondary to infectious etiology. Ten patients were started on steroids after biopsy at a mean period of 10 days (5-20 days) with high dose IV steroids for 3 days followed by oral steroids for 3-4 weeks. Six of 10 patients in the steroid group had complete recovery, 2 of 4 patients with severe interstitial sclerosis on biopsy developed CKD, and the remaining 2 patients were dialysis dependent. In the conservative treatment group, 9 out of 17 (~50%) remained on dialysis. In another study by Muriithi et al, 114 out of 133 patients with AIN were given steroid therapy. Forty-nine percent of the patients treated with steroids showed full recovery, 39% partially recovered from AIN while 12% showed no improvement (13).

Few studies did not find any statistical significance in terms of the benefits of steroids for AIN, indicating the need for randomized studies. Clarkson et al compared the outcome between patients treated with corticosteroids (n = 16) versus conservative management (n = 26) for AIN. IV steroids started within 4 days of biopsy in the steroid group, whereas conservative treatment started within 3 days. Both of the groups were properly matched and outcomes were studied after 1 year follow-up (9). No significant difference was found in the outcome between the two groups in terms of median creatinine levels (*p* value = 0.4) during the follow-up time points of 1, 6, and 12 months after biopsy.

## 4. Conclusions

In conclusion, it is essential to consider AIN in the differential for unexplained AKI. Initial management should include conservative therapy by withdrawing any suspected causative agent. Renal biopsy is needed for confirmation in cases where kidney function fails to improve within 5–7 days on conservative therapy (11). A trial of corticosteroids can be started in biopsy-proven patients with AKI for <3 weeks. Steroid therapy is usually maintained for 4–6 weeks and dosage tapered over next 4 weeks (11).

Use of other immunosuppressives like oral or intravenous cyclophosphamide, cyclosporine, mycophenolate mofitil, and plasmapheresis have been considered rarely and proven beneficial for steroid-dependent or steroid-intolerant AIN (6,14). Risk of immunosuppression is very important to consider when giving steroids in patients with infection induced AIN, and steroids may have to be delayed until the active infection is completely controlled (2).

In our case, with the suspicion of infection periodontal abscess as the etiology the definitive diagnosis of AIN was made by the renal biopsy. The patient was first treated for the infection with abscess drainage and appropriate antibiotic coverage before starting on immunosuppression with 60 mg/day prednisone for 6 weeks. The steroid was dose tapered thereafter for 4 weeks and the patient showed complete recovery in renal function without any complications from both AKI and steroid use.

## Authors’ contribution

RR, SA, TC, LF, RN, NT have contributed to the primary draft. All authors have read the final version.

## Conflicts of interest

Authors have no conflict of interest.

## Funding/Support

No funding was obtained for the preparation of this manuscript.
